# Tracking and curating putative SARS-CoV-2 recombinants with RIVET

**DOI:** 10.1093/bioinformatics/btad538

**Published:** 2023-08-31

**Authors:** Kyle Smith, Cheng Ye, Yatish Turakhia

**Affiliations:** Department of Biological Sciences, University of California, San Diego, San Diego, CA 92093, United States; Department of Electrical and Computer Engineering, University of California, San Diego, San Diego, CA 92093, United States; Department of Electrical and Computer Engineering, University of California, San Diego, San Diego, CA 92093, United States

## Abstract

**Motivation:**

Identifying and tracking recombinant strains of SARS-CoV-2 is critical to understanding the evolution of the virus and controlling its spread. But confidently identifying SARS-CoV-2 recombinants from thousands of new genome sequences that are being shared online every day is quite challenging, causing many recombinants to be missed or suffer from weeks of delay in being formally identified while undergoing expert curation.

**Results:**

We present RIVET—a software pipeline and visual platform that takes advantage of recent algorithmic advances in recombination inference to comprehensively and sensitively search for potential SARS-CoV-2 recombinants and organize the relevant information in a web interface that would help greatly accelerate the process of identifying and tracking recombinants.

**Availability and implementation:**

RIVET-based web interface displaying the most updated analysis of potential SARS-CoV-2 recombinants is available at https://rivet.ucsd.edu/. RIVET’s frontend and backend code is freely available under the MIT license at https://github.com/TurakhiaLab/rivet and the documentation for RIVET is available at https://turakhialab.github.io/rivet/. The inputs necessary for running RIVET’s backend workflow for SARS-CoV-2 are available through a public database maintained and updated daily by UCSC (https://hgdownload.soe.ucsc.edu/goldenPath/wuhCor1/UShER_SARS-CoV-2/).

## 1 Introduction

Recombination is a known contributor to genetic novelty in SARS-CoV-2 (Jackson *et al.* 2021, Turakhia *et al.* 2022). Recent recombinant subvariants, such as XBB.1.5 and XBB.1.16, have been identified as among the most transmissible variants of SARS-CoV-2 seen so far (Yamasoba *et al.* 2023, Yue *et al.* 2023). Identifying and tracking recombinants in a timely manner is therefore critical to help public health officials promptly respond to emerging threats and implement appropriate control measures. Because of the epidemiological importance of recombinants, and because they violate the normal tree structure of evolution, a special naming convention (i.e. names starting with the letter “X”) is used for recombinant variants in Pango—the most widely used nomenclature system for SARS-CoV-2 variants (Rambaut *et al.* 2020). As of July 2023, ∼76 unique recombinant lineages (or ∼559, if including sublineages) have been formally identified under this system. However, several recent large-scale studies on SARS-CoV-2 recombinants have found hundreds to thousands of uniquely identifiable recombination events (Turakhia *et al.* 2022, Zhan *et al.* 2023), suggesting considerable underestimation in the Pango system of the actual prevalence of detectable recombination. This is possibly because of the significant manual effort that is involved in ratifying recombinants in the present system. First, volunteers are required to collect and present evidence of recombination that they found using the pangolin-designation GitHub Issues (https://github.com/cov-lineages/pango-designation/issues). Next, another group of experts laboriously review the proposals for potential issues (such as contamination or bioinformatic errors), before formally designating recombinant lineages that meet the quality standards—a process that often takes weeks to months. In this manuscript, we present RIVET, a tool that can significantly accelerate both aforementioned steps to designate recombinant lineages. In the future, RIVET may be combined with Autolin (McBroome *et al.* 2023) to fully automate the process of naming variants, including the special handling of recombinants. It may also help to build more accurate representations of the evolution of SARS-CoV-2 using phylogenetic networks and aid in the recombination analysis of other pathogens.

## 2 Results and discussion

**Figure 1.**
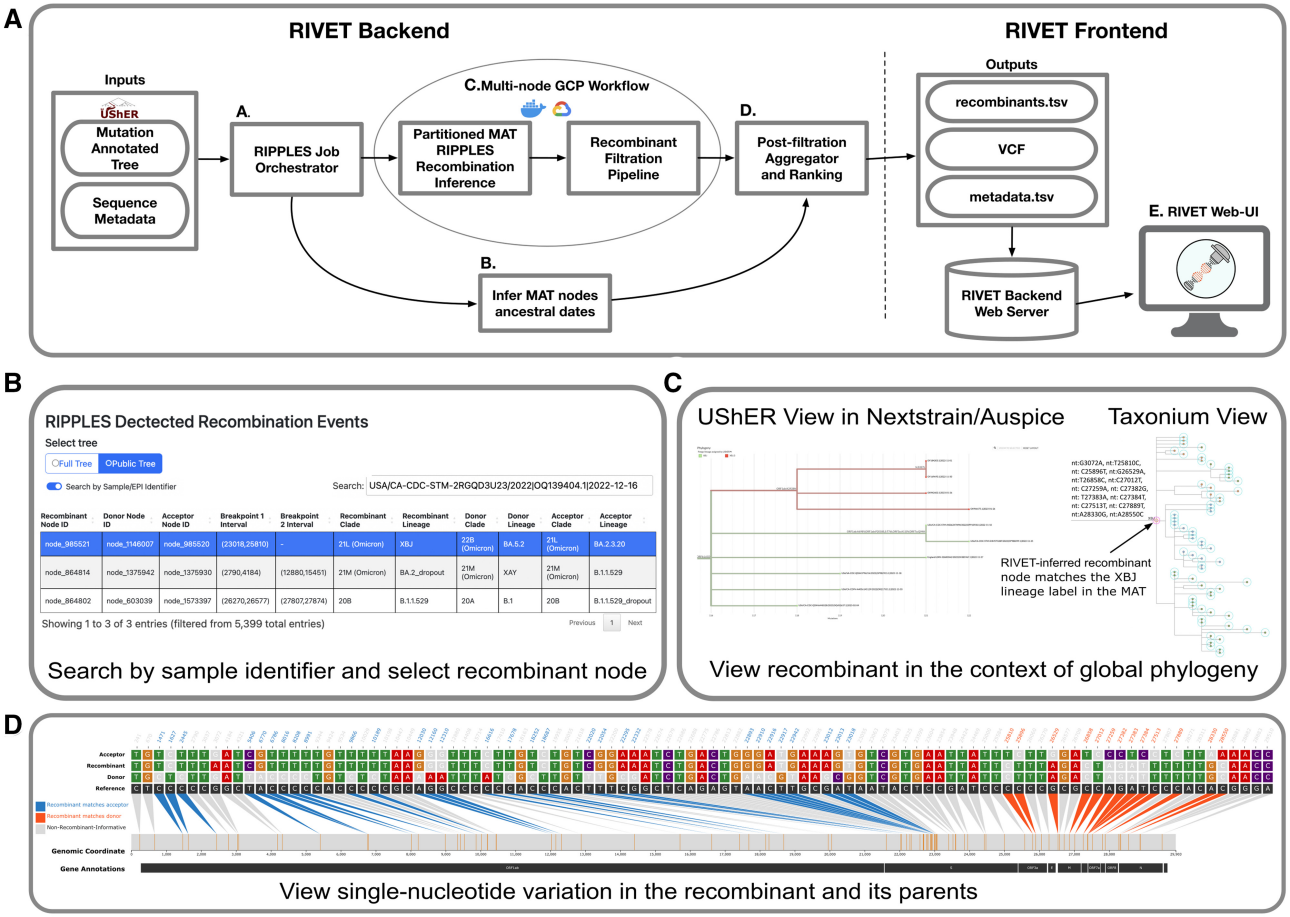
(A) An overview of RIVET’s backend and frontend components. (B) Search by sample/EPI identifier feature allows the user to search for recombinant ancestry in a given query sample. The detected recombinant results are filtered based on the query and node_985521 highlighted in blue has been selected by the user for further analysis. This node in the MAT was preannotated as the root of the XBJ lineage that was manually identified as recombinant by the Pango curation team (https://github.com/cov-lineages/pango-designation/issues/1268). (C) Nextstrain/Auspice and Taxonium view of the recombinant node descendants highlighting the selected recombinant node identified by the RIVET workflow in the 2023-07-02 public SARS-CoV-2 MAT and (D) its corresponding RIVET-based SNV plot.

Figure 1A shows an overview of the backend and frontend components of RIVET (Recombination Viewer and Tracker). RIVET’s primary aim is to significantly accelerate the process of identifying and curating SARS-CoV-2 recombinant lineages. In particular, we use RIVET’s backend to perform weekly analysis of SARS-CoV-2 recombination using the latest mutation-annotated trees (MATs) from UShER (McBroome *et al.* 2021, Turakhia *et al.* 2021) and host the results using RIVET’s frontend on a web interface (https://rivet.ucsd.edu/) for supporting the curation efforts. Similar to UShER, RIVET’s web interface also hosts separate results for two MATs: (i) a “full” MAT that includes sequences from GISAID (Shu and McCauley 2017), with restricted features to comply with GISAID’s usage terms and (ii) a “public” MAT that contains sequences only from unrestricted public databases. For each MAT, the web interface displays a table of all inferred trios of recombinant and parent (called donor and acceptor) nodes (Fig. 1B). The table also includes relevant information such as the inferred breakpoint intervals, parsimony score improvement resulting from the partial placement of recombinant sequences broken at breakpoints on parent nodes, current Pango lineage assignment of recombinant and parent nodes, 3SEQ-derived *P*-values of informative site sequence (Martin *et al.* 2010), date of origin of the recombinant, growth score, and so on. The table also displays potential quality issues for recombinants identified by RIVET’s filtration pipeline and can be searched and sorted using any field. Notably, the interface allows users to search for known or suspicious recombinant sequences using their sample/EPI identifiers or lineage designations (Fig. 1B). The table also links to UShER (Fig. 1C and Supplementary Methods), which can display a subtree of descendant recombinant sequences in the Auspice interactive phylogeny viewer (Hadfield *et al.* 2018). For the public tree, the table also provides a link to a Taxonium/Treenome view (Sanderson 2022, Kramer *et al.* 2023) for each recombinant, in which the phylogenetic contexts and genotype information of the recombinant and its parent sequences can be simultaneously visualized (Fig. 1C andSupplementary Methods). The frontend also provides a view of single-nucleotide variation (SNV) in the three sequences (each recombinant and its parents), with informative sites highlighted (Fig. 1D). This view draws inspiration from snipit (https://github.com/aineniamh/snipit) and uses the VCF produced by RIVET’s backend as input (Supplementary Methods). Additionally, RIVET’s frontend code can be used in a local HTTP server to visualize the recombinant-informative sites and breakpoint intervals for any generic set of sequences, including from other pathogens (instructions can be found on our documentation website).

RIVET’s backend (Fig. 1A) consists of a workflow that produces a set of output files that can be loaded to its frontend (Supplementary Methods). The workflow starts by comprehensively searching for potential recombinants from an input MAT using a newly optimized implementation of the RIPPLES software (Turakhia *et al.* 2022) that achieves 1–2 orders of magnitude speedup relative to the original implementation while producing identical results (Supplementary Methods). The key performance optimizations to RIPPLES include (i) amortizing computations of parsimony improvement for different breakpoint intervals, (ii) improving the memory locality of the algorithm, and (iii) achieving fine-grained parallelism through vectorized instructions (Supplementary Methods). Subsequently, the workflow initiates an automated pipeline that analyzes each RIPPLES-inferred recombinant in the MAT using raw genome sequences provided to the pipeline as input for potential quality issues that may have resulted from bioinformatic, contamination, or other sequencing errors. This quality control and filtration pipeline was developed and described previously in the RIPPLES manuscript (Turakhia *et al.* 2022). The workflow also infers the dates of origin of RIPPLES-inferred recombinant nodes in the MAT using sequence metadata and Chronumental (Sanderson 2021). This helps to rank emerging recombinants of epidemiological interest using an *ad hoc* growth score which is computed based on the recency of the recombinant ancestor’s Chronumental-inferred origin date and the growth of its specific set of descendant samples (Supplementary Methods). RIVET’s workflow has been heavily optimized for efficient and parallel execution on the Google Cloud Platform (Supplementary Methods). For example, on a MAT dated 2 July 2023, consisting of >15.3 million SARS-CoV-2 sequences, the workflow produced results within 2.25 h and with less than $4 in compute costs (Supplementary Table S1). We therefore do not expect compute costs to be a major constraint to frequently updating RIVET results.

RIVET analysis of a full MAT dated 2 July 2023, containing 15.3 million sequences, revealed 3665 unique recombinants, of which 847 passed all quality checks. The breakpoint distribution of high-quality recombinants that passed all automated quality checks showed increased recombination rates in the 3′ portion of the SARS-CoV-2 genome (Supplementary Fig. S1), consistent with the previous study (Turakhia *et al.* 2022). Intralineage recombination, which gets frequently overlooked, accounted for 30.3% of high-quality recombinants. RIVET inferences, such as recombinant-informative mutations and lineages of parent sequences, of known recombinants were largely consistent with those of manual curators (Fig. 1C and Supplementary Fig. S2). In the future, we plan to extend RIVET’s backend capabilities to support recombination inference and genomic surveillance efforts for other pathogens as well.

## Supplementary Material

btad538_Supplementary_Data
