# Supplementary material for: Tracking and curating putative SARS-CoV-2 recombinants with RIVET
Source: Bioinformatics. 2023 Aug 31;39(9):btad538. doi: 10.1093/bioinformatics/btad538 (PMC10493179; doi:10.1093/bioinformatics/btad538)
Supplement: btad538_Supplementary_Data [file btad538_supplementary_data.pdf]

# Supplementary Methods

## Optimizations and parameter settings in the RIPPLES software

The RIVET backend uses a refactored implementation of RIPPLES, called `ripples-fast`, which achieves 1–2 orders of magnitude speedup relative to the original implementation while producing identical results. The key performance optimizations include (i) amortizing computations of parsimony improvement for different breakpoint intervals, (ii) improving memory locality of the algorithm, and (iii) achieving fine-grained parallelism through vectorized instructions. (i) For every node in the mutation-annotated tree (MAT), `ripples-fast` starts by maintaining a mutation count vector that stores the number of sites along different positions in the genome at which the putative recombinant node differs from the reference. When performing partial placements, this mutation count vector is directly used to find the parsimony score improvement for each possible breakpoint interval. This eliminates the cost of traversal from root to that node in the mutation-annotated tree (MAT) to find corresponding mutations at that node. (ii) Since a single vector needs to be accessed at each node to find the parsimony score improvement, this technique of using a mutation count vector per node also improves the memory locality of the RIPPLES algorithm. (iii) Finally, because recombinants are relatively rare, `ripples-fast` utilizes the SSE-based vector instructions available on Intel Processors to test for the presence of putative recombination at multiple breakpoints in parallel. This is in addition to the multithreaded and multiprocess parallelism that was already available in RIPPLES.

By default, RIVET uses sensitive search parameters for RIPPLES (i.e, setting `--branch-length 3 --num-descendant 5 --parsimony-improvement 3`). These parameters require that a node have a branch length of at least 3 mutations and a minimum of 5 descendant tips to be considered for recombination. Additionally, the partial placement parsimony score should improve by at least 3 mutations for a node to be flagged as a potential recombinant.

## Estimating date of origin of recombinants and growth scores

Since recombinants discovered through RIPPLES correspond to internal nodes of the MAT, their origin or sampling date is not directly available through sequence metadata. However, if the sequence metadata, which contains the sampling date of each sequence in the MAT, is provided as input by the user, RIVET also launches a parallel Chronumental process (Sanderson, 2021) to build a time tree from the MAT. On RIVET's frontend interface, users can sort the recombinant list based on the origin date to quickly review recombinants that have been inferred to have emerged recently.

Additionally, to help prioritize emerging recombinants of epidemiological interest for the purposes of recombinant lineage identification and tracking, RIVET assigns each detected recombinant a growth score and outputs a ranked list of putative recombinants. The recombinant growth metric below, G(*R*), for a recombinant node with a set of descendants *S* is defined below:

$$G(R) = 2^{-m(R)}. \sum_{s \in S} 2^{-m(s)}$$

In the equation above, $m(R)$ and $m(s)$ correspond to the number of months (30-day intervals)

elapsed since the recombinant node $R$ was inferred to have originated and its descendant sequence $s$ was sampled, respectively. The growth score above, $G(R)$, is computed for each detected recombinant $R$, and the final recombinant list is ranked based on descending growth scores.

### Efficient RIVET workflow parallelization on the Google Cloud Platform (GCP) and output files

The entire RIVET backend pipeline is contained within a public Docker image that can be massively parallelized across multiple servers on Google Cloud Platform (GCP). In a YAML configuration file provided, the user can specify the number of instances and machine type to run the RIVET job. By default, we run the workflow on two `n2d-highcpu-32` instances. Upon initiating, RIVET loads the input mutation-annotated tree (MAT) and conducts a parallel search for long-branches that will be considered for the recombination search. The number of long branches is then automatically partitioned uniformly across the specified number of GCP instances. Each GCP instance searches its range of long branches in parallel for recombination events. Immediately upon completion of the search phase, an automated filtration pipeline begins on the instance to check for potential sequencing and bioinformatic quality issues with each detected recombinant. Once every GCP instance has completed both the search and filtration steps, RIVET aggregates the results from each instance locally, and ranks the recombinant results.

### RIVET's frontend implementation details

The RIVET frontend is a Flask application (Grinberg, 2018) that loads and pre-processes the output files generated by RIVET's backend, which includes a tab-delimited recombinant results file, a VCF file containing all the single-nucleotide variants (SNVs) of the trio sequences (recombinant, donor, acceptor) and a tab-delimited descendants file containing a mapping of all trio node ids to their respective set of descendants. RIVET utilizes *cyvcf2* (Pedersen and Quinlan, 2017), which is a Python library wrapper around *htslib* (Bonfield *et al.*, 2021), to enable fast parsing of the input trio VCF file. The RIVET web interface displays the recombination results ranked by growth score in a table format where each row in the table is a detected recombinant. To see the SNVs for a particular recombinant of interest, the user can select a row to dynamically render an interactive visualization built using *d3.js* that displays the SNVs for the selected recombinant and its two parents, with respect to the SARS-CoV-2 reference (GenBank MN908947.3, RefSeq NC_045512.2). The plot shows all positions where at least one of the trio sequences contains a variant, however the recombinant-informative sites are highlighted where the recombinant matches the donor or the acceptor sequence, for clear visualization of the inferred breakpoint intervals. By clicking the available buttons, any view of the visualization can be downloaded in SVG format, for high-quality publication-ready figures, or copied and pasted directly into lineage proposal GitHub Issues, for example. The SNV visualization also contains several built-in interactive features, such as the ability to query and download the descendants specific to a particular node in the trio by clicking the corresponding track label. RIVET's web interface provides integration with phylogeny viewer tools, namely Nextstrain's Auspice (Hadfield *et al.*, 2018) and Taxonium's Treenome browser (Sanderson, 2022; Kramer *et al.*, 2023). To generate the Nextstrain Auspice view, RIVET selects a random single subtree (default parameter is a subtree containing 10 descendants) from the MAT and queries the UShER web API (UShER.bio) to return its corresponding subtree that can be viewed in Auspice. For the Taxonium view, RIVET queries the Taxonium web API with the

selected recombinant and its parental sequences using a custom-built Taxonium JSONL configuration file that is produced as an output of RIVET's backend pipeline using `taxoniumtools` (https://github.com/theosanderson/taxonium). The configuration file helps highlight the selected trio of sequences in the global phylogeny and color the tips of the phylogeny by their Pango lineage classification annotated in the MAT.

## Supplementary Tables

**SupplementaryTable 1:** RIVET backend runtime and cost estimates using two `n2d-highcpu-32` instances on the Google Cloud Platform (GCP) for different MATs.

| Date (type) of MAT | Number of sequences in the MAT | Number of unique recombinants discovered | | Ripples-fast runtime | Total runtime (including QC, ranking etc.) | Estimated compute cost |
|---|---|---|---|---|---|---|
| | | Pre-filter | Post-filter | | | |
| October 31, 2022 (Public) | 6,427,951 | 1,413 | 421 | 20m 22s | 45m 25s | $2.06 |
| November 30, 2022 (Public) | 6,497,825 | 1,452 | 441 | 20m 19s | 49m 17s | $2.23 |
| December 31, 2022 (Public) | 6,612,971 | 1,473 | 455 | 17m 01s | 43m 10s | $1.97 |
| January 31, 2023 (Public) | 6,716,605 | 1,470 | 453 | 23m 35s | 51m 10s | $2.34 |
| July 2, 2023 (Public) | 7,667,784 | 1,727 | 426 | 25m 55s | 57m 21s | $2.39 |
| July 2, 2023 (Full) | 15,360,149 | 3,665 | 847 | 94m 43s | 135m 47s | $3.68 |

## Supplementary Figures

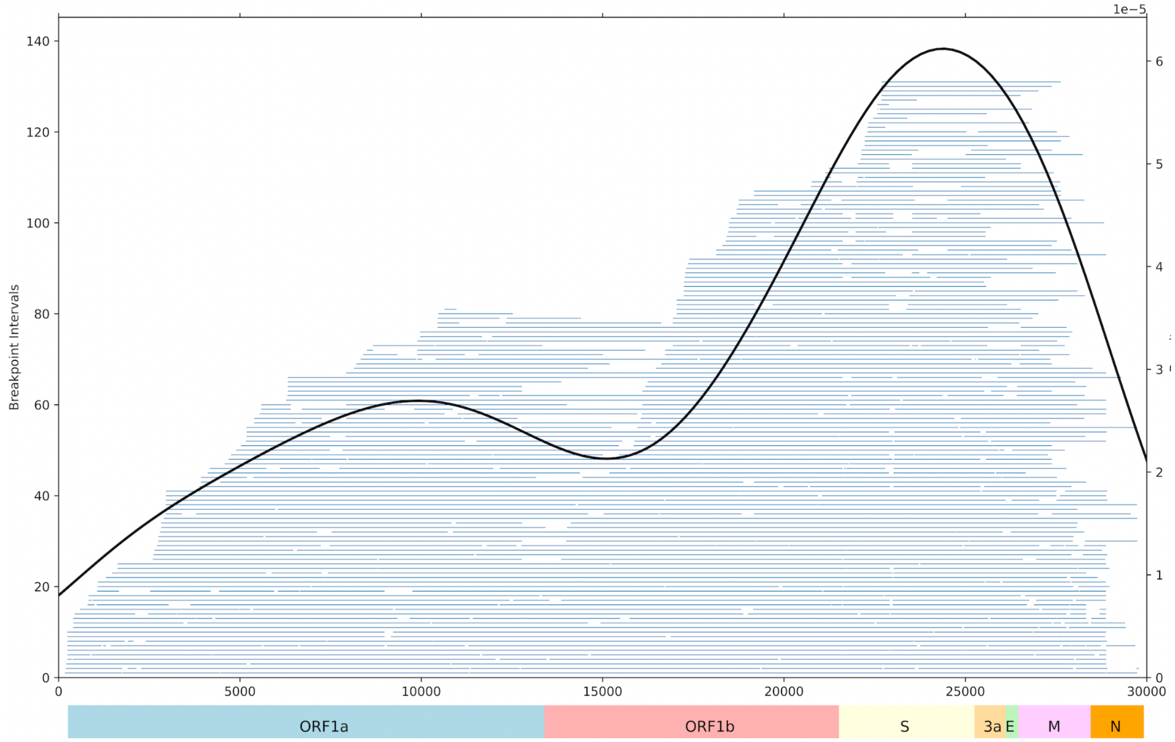


**Figure S1:** Breakpoint intervals (in blue bars) of recombinants that passed all quality filters along with a smoothed density plot of their midpoints (in black).

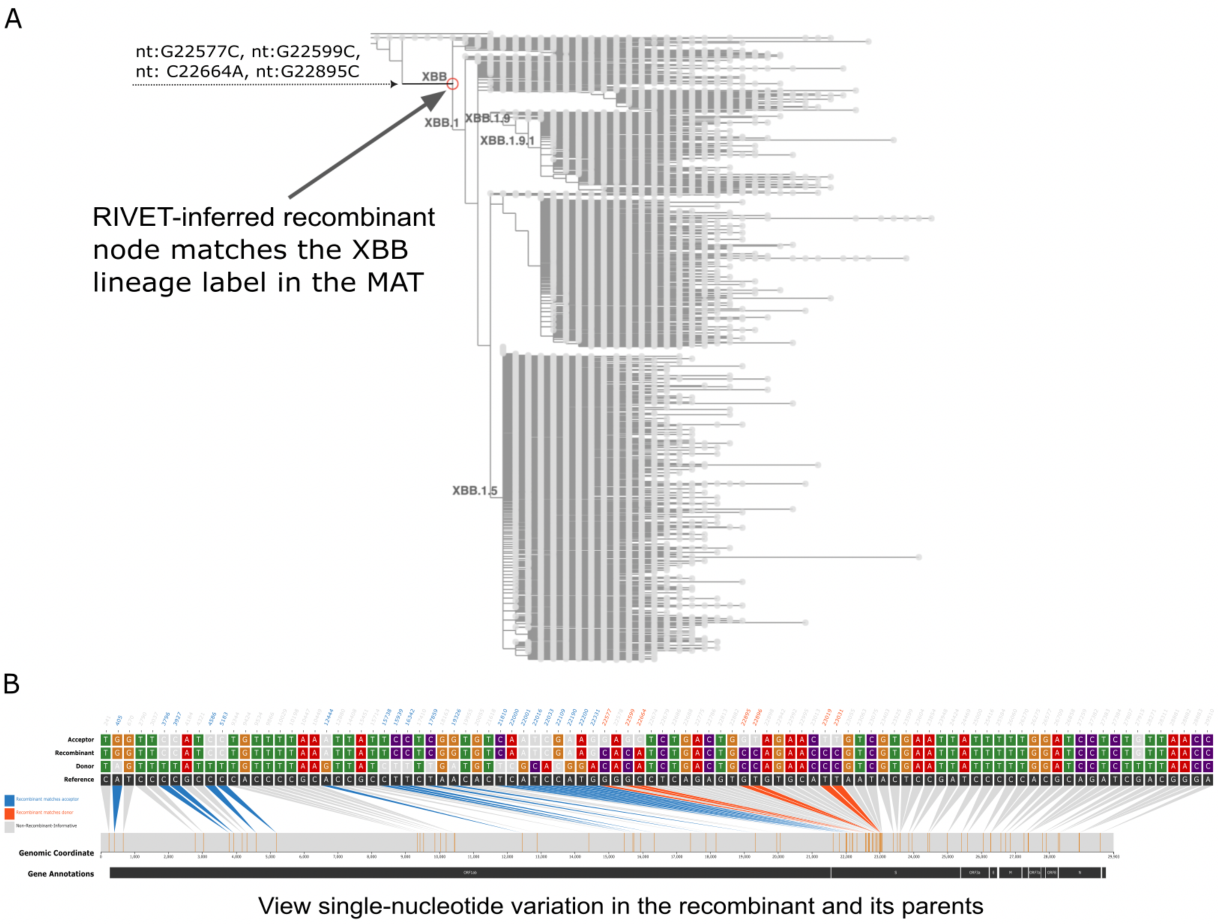


**Figure S2:** (**A**) Taxonium view of the 2023-07-02 public SARS-CoV-2 MAT highlighting the node that is pre-annotated as the XBB root by the Pango curation team (https://github.com/cov-lineages/pango-designation/issues/1058) that matches a RIVET-inferred recombinant node, (**B**) its corresponding RIVET-based SNV plot.

## Supplementary References

Bonfield,J.K. *et al.* (2021) HTSlib: C library for reading/writing high-throughput sequencing data. *GigaScience*, **10**, giab007.

Grinberg,M. (2018) Flask Web Development: Developing Web Applications with Python O'Reilly Media, Inc.

Hadfield,J. *et al.* (2018) Nextstrain: real-time tracking of pathogen evolution. *Bioinformatics*, **34**, 4121–4123.

Kramer,A.M. *et al.* (2023) Treenome Browser: co-visualization of enormous phylogenies and millions of genomes. *Bioinformatics*, **39**, btac772.

Pedersen,B.S. and Quinlan,A.R. (2017) cyvcf2: fast, flexible variant analysis with Python. *Bioinformatics*, **33**, 1867–1869.

Sanderson,T. (2021) Chronumental: time tree estimation from very large phylogenies.

Sanderson,T. (2022) Taxonium, a web-based tool for exploring large phylogenetic trees. *eLife*, **11**, e82392.
